# Supplementary material for: Scrub and murine typhus seroprevalence among blood donors in Laos
Source: PLoS Negl Trop Dis. 2026 Mar 10;20(3):e0014056. doi: 10.1371/journal.pntd.0014056 (PMC12974883; doi:10.1371/journal.pntd.0014056)
Supplement: Attachment — Submitted filename: PNTD_seroprevalence_response to reviewer_170226WP.docx [file pntd.0014056.s008.docx]

**Response to Reviewers**

PNTD-D-25-02129

Title: Scrub and murine typhus seroprevalence among blood donors in Laos

PLOS Neglected Tropical Diseases

We thank the editor and all reviewers for their constructive and insightful comments. We have carefully revised the manuscript and addressed all comments point by point below, with responses shown in blue. Text highlighted in yellow in this response document indicates wording that was added or revised in the manuscript. Page and line numbers refer to the revised manuscript.

**Reviewers' comments:**

Reviewer's Responses to Questions

**Key Review Criteria Required for Acceptance?**
As you describe the new analyses required for acceptance, please consider the following:
**Methods**
-Are the objectives of the study clearly articulated with a clear testable hypothesis stated?
-Is the study design appropriate to address the stated objectives?
-Is the population clearly described and appropriate for the hypothesis being tested?
-Is the sample size sufficient to ensure adequate power to address the hypothesis being tested?
-Were correct statistical analysis used to support conclusions?
-Are there concerns about ethical or regulatory requirements being met?

Reviewer #1: The methods are generally clear and appropriate, but please note the following:
1. It is stated that "concentrations of 1:8000, 1:4000 and 1:2000 dilutions of antigen" were used to coat plates, although normally this would be expressed as a protein concentration. If the investigators are not measuring protein content, how do they standardise the coating process between different batches of antigen?

Response: Bacterial cell lysate antigens were prepared by isolation and sonication of intracellular bacteria from cell culture. As bacterial protein concentration could not be quantified directly, antigen coating was standardised by checkerboard titration of each new batch against well-characterised serum samples to determine the optimal working dilution.

We have added this explanation to the Methods section in the revised manuscript (now lines 154–157), which reads as follows: Intracellular bacteria were isolated from infected cell cultures and subjected to sonication to generate whole-cell lysate antigens. Because direct quantification of bacterial protein concentration was not feasible, antigen coating concentrations were standardized for each batch by checkerboard titration using well-characterized reference sera to determine the optimal working dilution.

2. The selection of samples per province was stratified by age and gender, but within these strata, was sample selection randomised and if so, how?

Response: We did not perform random selection within each age–gender stratum since young adults are over-represented in the blood donor population and we wanted a spread across different age groups. For each province, we selected 100 samples and attempted to include individuals across the widest possible age range (≥17 years). Within the available samples, we tried to ensure that different age groups were represented as much as possible.

We have clarified this in the Methods section (now lines 135-138) of the revised manuscript which read as follows: For the detection of IgG antibodies against the scrub typhus group (STG) and typhus group (TG), we selected 100 serum samples from each of twelve provinces in Laos (total = 1200).

Most blood donors are younger adults and random sampling was not possible. Selection of samples for this study was based on donor age and sample availability, to be representative of different age groups.

We have also discussed this in the limitations section of the Discussion (now lines 508-513) in the revised manuscript, which reads as follows:

Second, we selected 100 participants per province, which may not accurately reflect the true exposure risk in each region. In addition, samples were obtained from blood donors, resulting in underrepresentation of older individuals and limiting the upper age range of participants. As selection within age strata was not randomized, the final age distribution reflects the characteristics of the donor population rather than the general population. These factors may introduce selection bias, and the relatively small sample size per province contributed to wide confidence intervals for several odds ratios, indicating substantial uncertainty in these estimates.

3. Details of the fluorescent conjugate used for the IFA are not provided. Please include.

Response: We apologize for lacking of this information IFA was performed using goat anti-human IgG (Fc) FITC conjugate (SeraCare USA, KPL 02-10-20; cat. no. 5230-0291) and used at 1:100 in PBS containing 2% skim milk and 0.05% Evans blue counterstain.

We have added this detail in the Method section of revised manuscript (now lines 177-180) which read as follows“ If a sample had an ELISA IgG OD ≥ 0.5[8], subsequent IgG IFA was performed for the same rickettsia group, using the same antigens. Bound antibodies were detected using goat anti-human IgG (Fc)-specific fluorescein isothiocyanate (FITC) conjugate (SeraCare USA; KPL 02-10-20; catalog no. 5230-0291), diluted 1:100 in PBS containing skim milk and 0.05% Evans blue counterstain.

4. In the Introduction, it is stated that GIS methods would be used to identify disease hotspots. This doesn’t seem to fit with the actual statistical approach employed?

Response: We thank reviewer for highlighting this point. We agree that the term “ to identify disease hotspots” imply the use of formal spatial statistical analyses. In this study, GIS was used to visualize the geographic distribution of seroprevalence across province rather than to perform spatial cluster detection or hotspot statistical modelling.

We have revised the introduction to clarify this point (now lines 106-107) to read as “In addition, Geographic Information System (GIS) mapping was used to illustrate the spatial distribution of cases “

Ethics and regulatory requirements seem to be met.

Reviewer #2: Are the objectives of the study clearly articulated with a clear testable hypothesis stated? Yes
-Is the study design appropriate to address the stated objectives? Yes
-Is the population clearly described and appropriate for the hypothesis being tested? Yes
-Is the sample size sufficient to ensure adequate power to address the hypothesis being tested? The issues in sample size have been discussed by the authors.
-Were correct statistical analysis used to support conclusions? Yes
-Are there concerns about ethical or regulatory requirements being met? No

**Results**
-Does the analysis presented match the analysis plan?
-Are the results clearly and completely presented?
-Are the figures (Tables, Images) of sufficient quality for clarity?

Reviewer #1: The presentation of the results is generally of sufficient quality with the following small areas for improvement.

1. The narrative doesn't always refer to the correct panels for Fig. 2 (only A and B are mentioned).

Response: We have revised the manuscript to ensure that all relevant panels of Fig. 2 are correctly referenced in the text (lines 247-266) which read as follows

All donor samples (n = 1198) were screened using ELISA for STG and TG IgG antibodies. The overall median OD percentage for STG was 0.032 (0.015, 0.131), and for TG it was 0.046 (0.020, 0.162) (S1 Table). Donors with an ELISA OD ≥ 0.5 for STG accounted for 118 participants (10.20 %, 95% CI: 8.56–12.10), while 139 participants (12.10%, 95% CI: 10.30-14.10) had ELISA OD ≥ 0.5 for TG (S2 Table). We identified seven participants (0.58%), each of whom had an ELISA OD ≥ 0.5 for both STG and TG.

Among participants with an ELISA OD ≥ 0.5 for STG, when categorized by gender, we observed ELISA positivity in 11.91% of males (95% CI: 9.87–14.3) and 5.33% of females (95% CI: 4.48–8.09) (S2 Table and Fig 2A). The highest ELISA positivity for STG was found in participants aged 47 years or older (13%, 95% CI: 6.42-24.4), while the lowest was observed in those aged 25-30 years (8.46%, 95% CI: 5.7-12.40) (S2 Table and Fig 2C). When categorized by province, the highest ELISA positivity for STG was observed in Xiangkhuang (31%, 95% CI: 22.8–40.6), followed by Huaphan (30%, 95% CI: 21.90–39.60) and Oudomxay (22%, 95% CI: 15–31.10) (S2 Table and Fig 2E).

Similarly, for TG, ELISA positivity was observed in 11.66% of males (95% CI: 9.65–14.0) and 11.47% of females (95% CI: 8.63–15.10) (S2 Table and Fig 2B). The same age-related pattern was observed, with the highest positivity in participants aged 37-46 years (17 %, 95% CI: 13–22) and the lowest in those aged was aged equal or more than 17 to 24 years (4.66 %, 95% CI: 2.74–7.81) (S2 Table and Fig 2D). The top three provinces with the highest ELISA positivity for TG were Oudomxay (27%, 95% CI: 19.30–36.40), Attapue (20%, 95% CI: 13.30–28.90), and Vientiane Province (18%, 95% CI: 11.70-26.70) (S2 Table and Fig 2F)

2. The ODs are provided as medians and percentiles it seems, but in inconsistent format (using a comma separator or dash). For instance, "The overall median OD percentage for STG was 0.034 (0.017, 0.170), and for TG it was 0.046 (0.020-0.162)." It is only when reading the supplement that it is clear the numbers in parentheses are percentiles rather than ranges. This needs to be made explicit in the main text. Also, these appear to be OD values and not "percentages".

Response: We have amended the manuscript to address these points (lines 244-245)

3. The narrative highlights certain age groups as having a higher seroprevalence, but this isn't significant for either disease (or even close). So, it should simply be stated that seroprevalence was not associated with age bracket for either disease.

Response: We thank the reviewer for this important comment. We have revised the Results section to avoid emphasizing descriptive differences between age groups. While age-specific proportions are presented descriptively, logistic regression analyses demonstrate that age group was not significantly associated with seropositivity for either STG or TG. The text has been modified accordingly to ensure consistency between descriptive results and statistical inference in the revised manuscript (seropositivity estimated by IFA) (now lines 367-372 and 374-379) as follows:

For STG IgG seropositivity, the positivity rate was 8.87% in males and 3.73% in females (Fig 4A). STG IgG seropositivity by age group was 5.38%, 5.88%, 8.98%, 8.52%, and 7.41% for ages 17–24, 25–30, 31–36, 37–46, and ≥47 years, respectively (Fig 4B). By province, STG IgG seropositivity was 27% in Huaphan, 19% in Oudomxay, and 16% in Xiangkhuang. In contrast, no STG IgG antibodies were detected among participants from Salavan (0%) (Fig 4C and 4D).

For TG IgG seropositivity, the positivity rate was 5.87% in females and 3.28% in males (Fig 4A). TG IgG seropositivity by age group was 1.79%, 4.41%, 4.33%, 5.93%, and 3.70% for ages 17–24, 25–30, 31–36, 37–46, and ≥47 years, respectively (Fig 4B). By province, TG IgG seropositivity was 10% in Attapue and Oudomxay, 8% in Bokeo, and 4% in Luang Namtha, Luang Prabang, and Vientiane Province, with other provinces showing lower or no seropositivity. No TG IgG seropositivity was detected in participants from Huaphan (Fig 4C and 4E).

We have added the results of the logistic regression (now lines 390-391 and 399-400) as follows “

For the STG group (Fig 5 and S4 Table), males had higher odds of seropositivity than females, both unadjusted (OR 2.51, 95% CI: 1.44–4.69) and adjusted (aOR 2.44, 95% CI: 1.36-4.68). Age group was not significantly associated with STG seropositivity in either unadjusted or adjusted analyses. By province (reference: Vientiane Capital), Huaphan (OR: 36.62, 95% CI: 7.53–660.62; aOR: 35.96, 95% CI: 7.31–650.99), Oudomxay (OR: 23.22, 95% CI: 4.66–421.71; aOR: 21.87, 95% CI: 4.22–402.45), and Xiangkhuang (OR: 18.86, 95% CI: 3.73–343.90; aOR: 18.17, 95% CI: 3.54-333.04) showed the highest odds ratios. Odds ratios could not be calculated for Salavan due to the absence of positive cases.

For the TG group (Fig 5 and S5 Table), males had lower odds of seropositivity than females, both unadjusted (OR: 0.54, 95% CI: 0.31–0.98) and adjusted (aOR: 0.53, 95% CI: 0.29–0.96). Age group was not significantly associated with TG seropositivity. By province (compared with Vientiane Capital), Attapue (OR: 5.44, 95% CI: 1.39–36.04; aOR: 5.48, 95% CI: 1.38–36.54), Oudomxay (OR: 5.44, 95% CI: 1.39–36.04; aOR: 5.13, 95% CI: 1.23–35.31), and Bokeo (OR: 4.26, 95% CI: 1.03–28.72; aOR: 4.76, 95% CI: 1.13–32.43) had the highest odds. Odds ratios could not be calculated for Huaphan due to the absence of positive cases. Several odds ratios for both STG and TG had wide confidence intervals, indicating substantial uncertainty around these estimates.

4. The labels on the plots in Fig. 4 are faint, low resolution and poorly legible. Please improve.

Response: The figure 4 has been amended to increase the resolution (lines 334-353)

5. The panel label for Fig. 5D should read "adjusted".

Response: Fig 5D has been amended (lines 407-421)

Reviewer #2: Does the analysis presented match the analysis plan?- Yes
-Are the results clearly and completely presented? Yes
-Are the figures (Tables, Images) of sufficient quality for clarity? Yes

**Conclusions**
-Are the conclusions supported by the data presented?
-Are the limitations of analysis clearly described?
-Do the authors discuss how these data can be helpful to advance our understanding of the topic under study?
-Is public health relevance addressed?

Reviewer #1: The discussion and conclusions do a reasonable job, but could dig a bit deeper:
1. Are information available on any potential demographic biases among blood donors in Laos, in terms of things like socioeconomic status, education level, residence in more urbanised areas etc.? The authors hint at likely differences between ST/MT febrile patients and donors, but this could be explored a bit more. It seems unlikely that your average subsistence farmer is well represented among blood donors, for example.

Response:

We thank the reviewer for this insightful comment. We agree that blood donors are unlikely to be fully representative of the general population and may differ from febrile patients in important demographic and occupational characteristics. In particular, individuals engaged in subsistence farming or other agricultural work who may have higher exposure to scrub typhus may be underrepresented among blood donors.

Although we do not have detailed data on socioeconomic status, education level, or occupation to formally assess these differences, we acknowledge that such selection factors could influence the magnitude of seroprevalence differences observed between febrile patients and donors. While donor addresses were recorded, these may not reliably reflect long-term residence or exposure setting, as individuals may work or temporarily reside in different areas. Therefore, we were unable to accurately classify donors as urban or rural.

We have now expanded the Discussion (lines 486-494) We did not collect detailed data on socioeconomic status, education level, occupation, or residential classification; these factors may influence differences in seropositivity between febrile patients and blood donors. While donor addresses were recorded, these may not reliably reflect long-term residence or exposure settings, as individuals may work in different areas. In addition, residential classification (urban versus rural) was not systematically recorded for donors in the present study, limiting direct comparison of exposure setting with previous reports. Therefore, demographic and occupational selection bias among blood donors cannot be excluded

2. The origin of the samples during the peak of the Covid pandemic is mentioned as a possible limitation. Could the Covid-19 pandemic policies of Laos be briefly summarised here? If they were as restrictive as Vietnam's zero-Covid approach for example, the impact on exposure to ST/MT could have been very substantial for many months at least.

Response: We thank the reviewer for this important comment. Blood samples in this study were collected between January and August 2021. Laos initially implemented border closures in March 2020 following the first reported COVID-19 cases. A lockdown was introduced between March and May 2020, domestic case numbers remained low for much of 2020, and most economic activities within the country resumed, including domestic travel and restaurant operations. In April 2021, following the detection of a new case and community transmission, stricter movement restrictions were reintroduced. Therefore, our sampling period spanned both less restrictive months (January-March 2021) and periods of increased restriction (April-August 2021). We acknowledge that changes in mobility during this period may have influenced short term exposure risk. However, IgG antibodies against *O. tsutsugamushi* and *R. typhi* are known to persist for months to years after infection. Consequently, the seroprevalence observed in 2021 likely reflects cumulative past exposure rather than infections occurring solely during the pandemic restriction period. We have revised the Discussion section to clarify the timing of sample collection and COVID-related movement restriction in Laos.

We have added a bit of the regulation during COVID -19 in Laos in the revised manuscript in discussion section (lines 494-498) which read as follow: One possible explanation is that sample collection occurred during the COVID-19 pandemic, with blood collection between January and August 2021. During this period, tighter movement restrictions were implemented in April 2021 which may have temporarily influenced population mobility and short-term exposure. However, IgG antibodies can persist for at least 18 months in scrub typhus patients[13] and up to 12 months in murine typhus patients[9], showing higher antibody levels due to recent immune activation. In contrast, blood donors represent individuals with past exposure resulting in lower IgG titers. This difference in antibody persistence may explain the lower number of seropositive cases among healthy donors compared with patients. Therefore, short-term movement restrictions during part of 2021 are unlikely to fully account for the marked reduction observed. In addition, many areas in Vientiane Capital have become more urbanized over the past decade, leading to a shift in vector habitats toward rural areas and we also observed fewer scrub typhus cases in Mahosot Hospital.”

3. The gender differences in seroprevalence are barely touched upon. Are there comparable data for surrounding countries?

Response: We thank the reviewer for highlighting this point. Although there have been a few seroprevalence studies conducted in healthy populations in neighboring countries, including North Vietnam Nadjm B et al., 2021) and Thailand (Gonwong S et al., 2022), differences of seropositivity by gender were not described in the Vietnamese study.

In Thailand, a nationwide seroprevalence study of rickettsioses collected samples from four regions (central, north, northeast, and south). However, this study investigated antibodies only in young Thai men serving in the Royal Thai Army, most of whom were in the same age group (21 years old). Therefore, gender comparisons could not be assessed.

Regarding seroprevalence studies in patients who required a blood draw for any reason after consultation with a medical doctor, a study conducted in Myanmar showed a similar trend to our findings: scrub typhus seropositivity was higher in men than in women, whereas typhus group seropositivity was higher in women than in men.

We have now added this information to the Discussion section and provided possible explanations for these observed differences (lines 448-453) which read as follow:

Seroprevalence studies conducted in healthy populations in neighboring countries, including North Vietnam[12] and Thailand[13], did not report gender-specific differences in seropositivity. A study conducted in Myanmar among patients who required blood sampling after medical consultation [14] demonstrated a pattern similar to our findings: scrub typhus was higher in men, whereas murine typhus group seropositivity group seropositivity was higher in women.

4. It is stated that "However, no STG seropositive cases were detected in Salavan in our selected population. This finding was surprising as a previous study on the causes of nonmalarial febrile illness in Laos showed that 7% of patients were confirmed to have acute scrub typhus [1]." The age of the cited study should be highlighted here, as no doubt the economic/demographic changes in Laos in the past 10 - 20 years have been seismic.

Response:

We thank reviewer for this suggestion and we have added information about year of the cited study in the discussion (lines469-472) as follows: However, no STG seropositive cases were detected in Salavan in our selected population. This finding was surprising as a previous study on the causes of non-malarial febrile illness in Laos, conducted between 2008 and 2010, showed that 7% of patients were confirmed to have acute scrub typhus [1].

5. The conclusions state “...confirming that rickettsial infections are widespread and remain an important cause of febrile illness throughout the country”. I don't think this study can reach this conclusion, as it is examining exposure in healthy individuals some years ago (and during the pandemic). It can only conclude that exposure to both diseases seems substantial in healthy individuals, even during a period when exposure would have been expected to be impeded by movement restrictions.

Response: We thank reviewer for highlighting this point. The conclusion has been amended accordingly to read as “This study demonstrates substantial prior exposure to both STG and TG in multiple provinces of Laos, including during the COVID-19 period. However, these findings reflect past exposure in healthy individuals and do not directly quantify the current burden of febrile illness attributable to rickettsia infections “(lines 523-526)

Reviewer #2: Are the conclusions supported by the data presented? Yes
-Are the limitations of analysis clearly described? Yes
-Do the authors discuss how these data can be helpful to advance our understanding of the topic under study? Yes , I have added some comments under "Summary and General Comments"
-Is public health relevance addressed? Yes

**Editorial and Data Presentation Modifications?**

Use this section for editorial suggestions as well as relatively minor modifications of existing data that would enhance clarity. If the only modifications needed are minor and/or editorial, you may wish to recommend “Minor Revision” or “Accept”.

Reviewer #1: 1. In the method section "Blood donors", the sentence "Serum samples were collected at the National Blood Centre and stored at -30°C before being transferred to the Lao-Oxford-Mahosot Hospital-Wellcome Trust Research Unit (LOMWRU), where they were stored at -80°C," should end in a full stop.

Response: This has been amended accordingly (line 133)

2. In the legend for Fig. 3, replace "represent" with "represented".

Response: This has been amended accordingly (line 331)

3. The subtitle "Logistic regression seropositivity" in the Results should be "Logistic regression and odds of seropositivity".

Response: This has been amended accordingly (line 384)

4. "By province (reference: Vientiane Capital)” is repeated at the beginning of a sentence in the paragraph under the "Logistic regression seropositivity" subheading. (Lack of line numbers in the manuscript is making these corrections difficult to describe...!)

Response: The repeated word has been removed

5. The abbreviation "aOR" for adjusted odds ratio is not used consistently.

Response: This has been amended accordingly (lines 392-394 and lines 401-403)

6. At the beginning of the Discussion, "Rickettsia infection" should be "rickettsial infections".

Response: This has been amended accordingly (lines 435)

7. The manuscript refers to either 17 or 18 provinces of Laos in total. Please check the correct number.

Response: This has been to correct as 17 provinces (line 122)

Reviewer #2: I have added some comments under "Summary and General Comments".

**Summary and General Comments**

Use this section to provide overall comments, discuss strengths/weaknesses of the study, novelty, significance, general execution and scholarship. You may also include additional comments for the author, including concerns about dual publication, research ethics, or publication ethics. If requesting major revision, please articulate the new experiments that are needed.

Reviewer #1: This is an important study on the seroprevalence of scrub typhus and murine typhus among blood donors in Laos, providing a new perspective following some groundbreaking work on these infections in Laos that is now more than 10 years old. Generally, the manuscript is straightforward and interpreted appropriately, but there are some stylistic problems, minor methodological issues, and the discussion requires a bit more context. See specific points to be addressed in the boxes above.

The results part of the abstract is rather packed with statistical reporting, which alongside the use of abbreviations at the beginning of sentences, makes it a very tough read. It would be more useful to summarise the key findings, simply stating what was significantly different or not, without all the odds ratios and CIs being quoted. Ironically, one of the more interesting findings (the significant and opposite gender difference for each disease) is not even mentioned here.

Response: We thank reviewer for highlighting this point. The result section in abstract (lines 41-44) has been revised to read as

“Results

Overall seroprevalence was 7.26% (95%CI:5.93-8.87) for STG and 4.09% (95% CI:3.11-5.37) for TG. STG seroprevalence was highest in Huaphan (27%), Oudomxay (19%) and Xiangkhuang (17%), all in northern Laos. TG seroprevalence was 10% in both Oudomxay (north) and Attapue (south). Compared with Vientiane Capital, these provinces had significantly higher seropositivity. No significant association was observed with age group. STG seropositivity was higher in males, whereas TG seropositivity was higher in females.”

Reviewer #2: Comments on “Scrub and murine typhus seroprevalence among blood donors in Laos”
1. Abstract: “scrub typhus” instead of “scrub”.

Response: This has been amended accordingly (line 27)

2. Why were only scrub typhus and murine typhus selected? Why was seroprevalence to spotted fever rickettsia also not tested for? In many studies, spotted fever rickettsial infections are more prevalent than murine typhus.

Response: We thank the reviewer for highlighting this point. The primary objective of this study was to investigate seroprevalence in areas endemic for scrub typhus and murine typhus, as these infections are the main targets of our ongoing and planned antibiotic efficacy studies.

3. Geographically and ecologically, how would the authors describe Huaphan, Oudomxay and Xiangkhuang? These places have higher seropositivity than that is the capital city.

Response: We thank reviewer for emphasized this point. Huaphan, Oudomxay and Xiengkhuang were the three provinces with the highest STG seroprevalence. These northern provinces are predominantly mountainous and rural, with extensive forest cover and agricultural land use. Such environments are considered as suitable habitats for chigger vectors and rodent host as Vientiane Capital may have lower levels of forest exposure, which could partly explain the lower observed seroprevalence. However, there are currently no published data on the prevalence *of Orientia tsutsugamushi* in chigger mites from these provinces, and further entomological studies would be needed to confirm this ecological hypothesis.

We have added to the discussion in lines 458- 466: Huaphan, Oudomxay and Xiangkhuang were the three provinces with the highest STG seroprevalence. These northern provinces are predominantly mountainous and rural, with extensive forest cover and agricultural land use. Such environments are considered as suitable habitats for chigger vectors and rodent hosts, possibly contributing to higher transmission risk compared to more urban areas like Vientiane Capital and Luang Prabang, where STG seroprevalence was lower. However, there are currently no published data on the prevalence of *O. tsutsugamushi* in chigger mites from these provinces, and further entomological studies would be needed to confirm this ecological hypothesis.

4. No correlation with age. 17 to >47 years was tested.

Response: We thank the reviewer for this comment. Participants aged 17 to ≥47 years were included in the analysis. Age group was evaluated in logistic regression models; however, no statistically significant association between age and seropositivity was observed for either STG or TG in both unadjusted and adjusted analyses. We have added the simple sentence in the result section to read as “Age group was not significantly associated with STG seropositivity in either unadjusted or adjusted analyses (lines 390-391) and “Age group was not significantly associated with TG seropositivity (lines 399-400)

5. The difference between the current and previous reported seroprevalence is very striking. The hypothesis that COVID restrictions might have had role to play may be the most like explanation. The actual seroprevalece may be much more. This may be discussed in the paper.
Additionally, the previous study (12) had enrolled participants from urban and peri-urban locations around the capital area. Were the blood donors selected in the current study from urban or rural areas, is this information available?

Response: We thank reviewer for the important comment. We have expanded the Discussion to further address the substantial difference studies, the potential contribution of multiple factors including COVID-19 restrictions, and the limitation regarding residential classification of blood donors.

We have revised text in the Discussion (lines 486-494) as follows: A striking finding was that Vientiane Capital showed lower seroprevalence for both STG and TG compared to our previous report in 2010[15], which reported STG IgG at 20.3% and TG IgG at 20.6%. In the current study, STG and TG were detected in only 1% and 2% of participants, respectively. Several factors may explain the conflicting findings between this study and previous reports, including that blood donors may not represent the general population, as they are less exposed to reservoirs or vectors, have limited age diversity, and differ in exposure-related behaviors. We did not collect detailed data on socioeconomic status, education level, occupation, or residential classification, these factors may influence differences in seropositivity between febrile patients and blood donors. While donor addresses were record, these may not reliably reflect long-term residence or exposure settings, as individuals may work or temporarily in different areas. In addition, residential classification (urban versus rural) was not systematically recorded for donors in the present study, limiting direct comparison of exposure setting with the previous report. Therefore, potential demographic and occupational selection bias among blood donors cannot be excluded.

6. What could explain the higher seropositivity for MT in females? A few lines on this may be added to the Discussion.

Response:

We thank the reviewer for this important observation. The higher seropositivity for murine typhus among females may be related to differences in daily activities and patterns of peridomestic exposure. Murine typhus is typically associated with rodent–flea transmission in urban and peri-urban environments. In rural Lao settings, women may spend more time in or around the household environment, where exposure to rodents and their ectoparasites could occur. However, we did not collect individual-level data on household conditions, animal contact, or behavioural risk factors. Therefore, this explanation remains speculative and warrants further investigation. We have added a brief discussion of this point in the manuscript in the Discussion section (lines 439-445) as follows: An explanation for why murine typhus seropositivity was higher in females is that it is typically associated with peridomestic rodent–flea transmission in urban and peri-urban environments. This finding may reflect differences in patterns of household or peridomestic exposure; however, we did not collect individual-level data on behavioural or environmental risk factors, and this interpretation remains speculative. Further studies incorporating detailed exposure assessments are needed to clarify the underlying drivers of this gender difference.

7. These infections re sympatric in many areas. How many subjects had seropositivity for both infections?

Response: Although seven samples were ELISA-positive for both TG and STG, confirmatory testing by IFA demonstrated that none were positive for both infections. We have added this in the result section (now lines 381-382) to read as “Although seven samples were ELISA-positive for both TG and STG confirmatory testing by IFA demonstrated that none were positive for both infections”

We believe that the revisions address all comments raised and improve the clarity and robustness of the manuscript.
